# Study on the Internal Stress and Thermal Anisotropy in Magnesium Alloys Using a Thermal Elastic Viscoplastic Self-Consistent Model

**DOI:** 10.3390/ma16227097

**Published:** 2023-11-09

**Authors:** Xianyun Zhu, Huamiao Wang, Yunxin Wu

**Affiliations:** 1 Light Alloy Research Institute, Central South University, Changsha 410083, China; zhuxysoler@gmail.com; 2 School of Mechanical Engineering, Shanghai Jiao Tong University, Shanghai 200240, China

**Keywords:** magnesium alloy, crystal plasticity, thermal anisotropy, internal stress

## Abstract

A thermal elastic viscoplastic self-consistent model is utilized to examine the thermal stress induced by the thermal anisotropy of single crystals during heat treatments. This model considers temperature-dependent elastic constants and critical resolved shear stress associated with thermal dilation. Simulation results demonstrate that under cooling, the elastic lattice strain increases significantly when constrained compared to unconstrained cooling. The deformation mechanism observed under cooling with constraint resembles tension along the constrained direction at room temperature. Polycrystals offer more deformation mechanisms to accommodate thermal anisotropy compared to single crystals, resulting in lower applied stress at the constrained boundary. Among the various observed textures, the maximum amplitude of residual lattice strain follows the following order: rolled > extruded > random. Lower thermal anisotropy in the entire polycrystal structure leads to reduced internal stress. For a single crystal within aggregates, the {00.2} plane experiences tensile lattice strain, while the {10.0} and {11.0} planes undergo compressive lattice strain due to the greater contraction of single crystals along the <c> direction compared to the <a> direction during cooling.

## 1. Introduction

Magnesium (Mg) alloys are being considered as potential lightweight structure materials in transportation industry under the background of energy shortage due to their low density and high specific strength [1,2,3]. Challenges still occur restricting the wide utility of Mg alloys, such as strong mechanical anisotropy and low ductility [4]. The mechanical anisotropy arises from the asymmetric hexagonal close-packed (HCP) crystal structure, resulting in a limited number of slip systems that can be activated at room temperature. Each of these systems has a notably distinct critical resolved shear stress (CRSS). Specifically, the CRSS of a non-basal slip is several times higher than that of the basal slip at room temperature [5]. Twinning behavior serves as an additional deformation mechanism, enabling Mg alloys to accommodate plastic strain and increase their ductility. However, twinning behavior exhibits polarity and introduces tension–compression asymmetry, which results in a strong dependence of the mechanical response on the loading direction [6,7] and stress states [5,8].

Moreover, mechanical anisotropy can also arise from thermal anisotropy and thermal stress experienced during various heat treatments [9]. At the microscale, the difference in thermal expansion coefficient between the <c> axis and the <a> axis can potentially cause deformation incompatibility among polycrystal aggregates [10], thereby amplifying the deformation anisotropy. The microscale strain mismatch between grains with different orientations gives rise to intergranular stress (type II stress) [11]. At the macroscale, the heterogeneous loading condition, such as significant temperature gradient between the surface area and internal region during heat treatments, results in non-uniform deformation across the material. The macroscale thermal stress induced by this non-synchronous deformation is associated with macroscale residual stress (type I stress) [12], which represents the cumulative effect of internal stress over a long distance.

Internal stress, which is a fundamental mechanical response to applied loading conditions, relies on various deformation mechanisms [9,12,13,14]. These mechanisms can be influenced by factors such as texture feature [15,16,17], strain path [18,19,20], strain history [21,22,23], and temperature [24,25,26]. In situ neutron diffraction measurements and simulation results have shown that the evolution of internal strain corresponds to the activity of specific deformation mechanisms. A kink point in the internal strain curve is often associated with the activation of a particular deformation mechanism [9,12,13]. A strong wrought texture can generate inelastic strain during unloading along a specific direction, which is a result of internal stress. However, this phenomenon is not observed in materials with a weak texture. The profuse twinning and detwinning activities occurring in the strong wrought texture contribute to this inelastic strain generation [15]. When subjected to compressive loading–unloading along the transverse direction (TD), the inelastic strain is higher compared to that under tensile loading–unloading. This is due to the detwinning activity during unloading, which promotes the generation of inelastic strain [20]. The pre-strain history effect can alter the distribution of internal stress. For example, pre-tension along the rolling direction (RD) delays the activation of extension twinning until the applied stress exceeds the combination of extension twinning resistance and the internal stress [22]. Temperature can also influence the favorability of deformation mechanisms by altering the thermal activation barrier of the non-basal slip [26]. In summary, most studies on internal stress focus on deformation at room temperature. The theoretical analysis of internal strain mismatch and the evolution of internal stress induced by thermal anisotropy during temperature changes are still scarce in previous literature.

The self-consistent (SC) scheme [27,28] has been widely recognized as a powerful tool for analyzing the underlying deformation mechanisms under different loading conditions. It is particularly useful in predicting anisotropic internal strains on crystalline planes, which have been shown to agree well with in situ neutron diffraction measurements [9,12]. The SC scheme considers each grain as a spherical inclusion embedded into an infinite homogeneous effective medium (HEM). The stress within each grain is calculated iteratively using an interaction law that takes the entire HEM into account. Meanwhile, a traditional alternative approach for calculating thermal stress is the finite element method (FEM) [29], which involves solving partial differential equations. FEM is widely utilized for predicting quench-induced residual stress [30,31,32], including a few studies on Mg alloys [33,34,35]. Previous FEM-based studies mainly focused on residual stress arising from temperature gradients and rarely addressed the anisotropic residual stress inherited from the asymmetric structure of HCP or the asymmetric thermal properties.

One of the major strengths of the SC scheme is its ability to robustly and efficiently solve the grain stress response to the applied boundary conditions for HCP materials, providing precise mesoscale deformation features, such as slip activities and texture evolution [36,37]. However, a limitation of the SC scheme is that it does not directly account for grain topology and local strain gradients [38,39], unlike the mesh structure-based aggregate representation in the FEM approach. In realistic materials with large dimensions undergoing rapid cooling, a pure SC model may not be capable of directly handling macroscale type I internal stress. Conversely, the FEM approach can predict this type of stress by tracing the difference of thermal expansion between the surface and the interion. However, the SC model can still be a valuable tool for theoretically investigating the intrinsic internal stress response of a material point to a homogeneous thermal boundary condition, which is the primary focus of the current study. By analytically understanding the intergranular stress among a material point without considering dimension effects, we can gain insight into the essence of plastic anisotropy and thermal anisotropy under heat treatments.

The current work is aimed at understanding the impact of thermal anisotropy on plastic anisotropy and internal strain mismatch in single crystals. This is achieved by utilizing a modified elastic viscoplastic self-consistent (EVPSC) model. The EVPSC-TDT, developed by H. Wang [40,41], represents a significant branch based on the widely used viscoplastic self-consistent model (VPSC) originally established by R. Lebensohn and C. Tomé [36,42]. The special goals of this work are as follows: (i) To elucidate the disparities of internal strain generation between polycrystal aggregates and single crystals subjected to cooling with constraint along one direction. Achieving this target will contribute to a better understanding of the response of internal strain distribution to a displacement boundary; (ii) To clarify the variances in internal strain generation caused by different texture features under unconstrained cooling conditions. These objectives aim to provide insights into the combined effects of thermal anisotropy and texture features on the generation of internal strains.

## 2. Methodology

**Figure 1:**
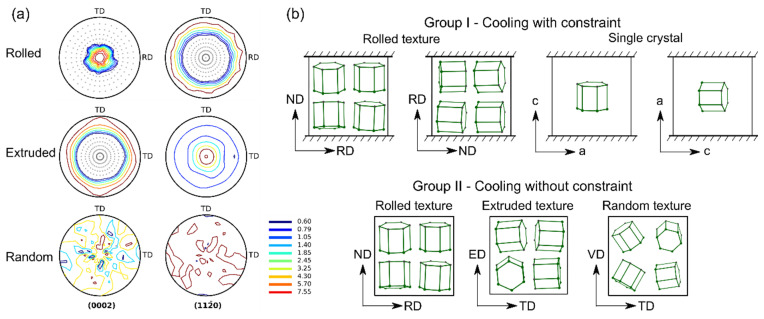
Three types of texture used in the numerical study (**a**) and the schematic diagram of two loading groups (**b**).

In this study, the simulations incorporate three different texture types of the AZ31B (Mg-3Al-1Zn) Mg alloy, which can be categorized into two groups, as shown in Figure 1. The three types of textures include rolled, extruded, and random textures (Figure 1a). The rolled texture contains 3000 grains, with the majority of c-axes aligned parallel to the normal direction (ND). The extruded texture is discretized into 1944 grains, where most of <a> axes are parallel to the extrusion direction (ED) and <c> axes are distributed uniformly and symmetrically around the ED. The third type of texture comprises 1000 grains with randomly orientated axes, with the vertical direction (VD) perpendicular to the paper.

The study employs two distinct groups of loading conditions, namely Group I and Group II, as depicted in Figure 1b. In Group I, the loading condition involves cooling with one direction constrained for both the rolled texture and the single crystal. For the rolled texture, fixed boundaries are imposed along the ND and the RD during the cooling process, respectively. Similarly, the single crystal sample undergoes cooling with fixed boundaries along the <c> axis and the <a> axis, respectively. In Group II, the loading condition entails cooling without any constraints for the rolled, extruded, and random textures. In this case, the tested texture samples deform freely without external interference during the cooling process. Consequently, when polycrystals have different orientations, the only driving factor for strain mismatch and strain accommodation between grains is the grain contraction induced by cooling. To explore various scenarios and refer to the practical cooling process [35], different initial cooling temperatures ($T_{ini}=573\;K$ and 773 K) and cooling rates ($T_{rate}=-20\;K/s$ and −100 K/s) are set until reaching the room temperature (298 K) at the end of each simulation.

## 3. Modeling

In the present study, a modified version of EVPSC model is employed with consideration of thermal effect. Based on the original EVPSC-TDT model [40,41], thermal strain is introduced into the framework. This accounts for the changes in lattice spacing caused by temperature variations. Additionally, the modified EVPSC-TDT model considers the temperature dependence of elastic constants and slip resistance. As temperature changes, the elastic constants and the slip resistance (the resistance to dislocation motion within the crystal lattice) are adjusted accordingly to capture the temperature-dependent behavior of the material. These specific changes in the EVPSC-TDT model allow for a more comprehensive and accurate description of the thermal effects on crystal plasticity behavior in the simulations conducted in this study.

The total strain rate of a single crystal $\dot{\varepsilon }$ consists of three components: elastic strain rate ${\dot{\varepsilon }}^{e}$, thermal strain rate ${\dot{\varepsilon }}^{t}$, and plastic strain rate ${\dot{\varepsilon }}^{p}$:

(1) $$\dot{\varepsilon }={\dot{\varepsilon }}^{e}+{\dot{\varepsilon }}^{t}+{\dot{\varepsilon }}^{p}.$$

The elastic constitutive law of a crystal is given by

(2) $${\dot{\sigma }}^{\nabla *}=L:\;{\dot{\varepsilon }}^{e}-\sigma tr({\dot{\varepsilon }}^{e}+{\dot{\varepsilon }}^{t}),$$

where ${\dot{\sigma }}^{\nabla *}$ is the Jaumann objective stress rate, which can be calculated by the elastic lattice spin rate tensor $w^{e}$, L is the fourth order elastic stiffness tensor, and σ is the Cauchy stress tensor.

Thermal strain rate ${\dot{\varepsilon }}^{t}$ is induced by temperature rate $\dot{T}$. The relation is given by

(3) $${\dot{\varepsilon }}^{t}=\alpha^{t}\dot{T},$$

where $\alpha^{t}$ denotes the thermal dilation tensor, which is composed of thermal expansion coefficient along *c*-axis $\alpha_{c}$ and along *a*-axis $\alpha_{a}$:

(4) $$\alpha^{t}=\left[\begin{array}{ccc} \alpha_{a} & 0 & 0\\ 0 & \alpha_{a} & 0\\ 0 & 0 & \alpha_{c} \end{array}\right].$$

The plastic strain rate ${\dot{\varepsilon }}^{p}\;$is given by

(5) $${\dot{\varepsilon }}^{p}=\sum_{\alpha }{\dot{\gamma }}^{\alpha }P^{\alpha },$$

where ${\dot{\gamma }}^{\alpha }$ is the strain rate of system α and $P^{\alpha }$ is the symmetric part of the Schmid tensor, which can be calculated by slip direction $s^{\alpha }$ and slip plane normal $n^{\alpha }$: $P^{\alpha }=\frac{1}{2}(s_{i}^{\alpha }n_{j}^{\alpha }+s_{j}^{\alpha }n_{i}^{\alpha })$.

Strain rate ${\dot{\gamma }}^{\alpha }$ can be calculated by a rate-dependent formula:

(6) $${\dot{\gamma }}^{\alpha }={\dot{\gamma }}_{0}{\left|\frac{\tau^{\alpha }}{\tau_{c}^{\alpha }}\right|}^{\frac{1}{m}}sgn(\tau^{\alpha }),$$

where $\tau_{c}^{\alpha }$ and $\tau^{\alpha }$ are the CRSS and resolved shear stress (RSS) of system α, respectively, and m is a rate sensitivity constant.

In the self-consistent scheme, each grain is considered as a spherical inclusion embedded within an HEM that represents the polycrystal aggregates. The relationship between total strain and stress for an inclusion and the HEM can be given by the following equations, respectively:

(7) $$\dot{\varepsilon }=M^{e}:\dot{\sigma }+M^{v}:\sigma +\alpha^{t}\dot{T}+{\dot{\varepsilon }}^{0},$$

(8) $$\dot{E}={\bar{M}}^{e}:\dot{\Sigma }+{\bar{M}}^{v}:\Sigma +{\bar{\alpha }}^{t}\dot{T}+{\dot{E}}^{0},$$

where $\dot{\varepsilon }$, σ, $M^{e}$, $M^{v}$, $\alpha^{t}$, ${\dot{\varepsilon }}^{0}$ are total strain rate, Cauchy stress, elastic compliance tensor, viscoplastic compliance tensor, thermal dilation tensor, and back-extrapolated strain rate for the grain, respectively. $\dot{E}$, Σ, ${\bar{M}}^{e}$, ${\bar{M}}^{v}$, ${\bar{\alpha }}^{t}$, ${\dot{E}}^{0}$ are the corresponding parts for the HEM.

The interaction between the grain and the HEM associating the strain and stress can be given by

(9) $$\dot{\varepsilon }-\dot{E}=-{\tilde{M}}^{e}:\left(\dot{\sigma }-\dot{\Sigma }\right)-{\tilde{M}}^{v}:(\sigma -\Sigma ),$$

where ${\tilde{M}}^{e}$ and ${\tilde{M}}^{v}$ are interaction tensors for the elastic part and viscoplastic parts, respectively. These two tensors can be further described by the elastic and viscoplastic Eshelby tensors, $S^{e}$ and $S^{v}$, as follows:

(10) $${\tilde{M}}^{e}={\left(I-S^{e}\right)}^{-1}:S^{e}:{\bar{M}}^{e},$$

(11) $${\tilde{M}}^{v}={\left(I-S^{v}\right)}^{-1}:S^{v}:{\bar{M}}^{v}.$$

The thermal dilation tensor for polycrystals (${\bar{\alpha }}^{t}$) can be self-consistently solved by P. Turner and C. Tomé [9,10]:

(12) $${\bar{\alpha }}^{t}={\bar{M}}^{e}{\langle {\left({M^{e}}^{-1}+{{\tilde{M}}^{e}}^{-1}\right)}^{-1}\rangle }^{-1}\langle {\left({M^{e}}^{-1}+{{\tilde{M}}^{e}}^{-1}\right)}^{-1}{M^{e}}^{-1}\alpha^{t}\rangle .$$

The temperature-dependent elastic constants $C_{ijkl}$ are defined following the formulation proposed by Varshni [43]:

(13) $$C_{ijkl}=C_{ijkl}^{0}-\frac{p_{ijkl}}{\exp\left(\frac{q_{ijkl}}{T}\right)-1.0},$$

where $p_{ijkl}$ and $q_{ijkl}$ are constants governing the rule of temperature dependence. $C_{ijkl}^{0}$ denotes the limit of elastic constant. T stands for the current temperature in Kelvin.

The basal slip and extension twinning are treated as temperature independent [24,25,44,45], the non-basal slips are given a temperature-dependent form similar to that proposed by Beyerlein and Tomé [46]:

(14) $$\tau_{cr}^{\alpha }=\left\{\begin{array}{cc} \tau_{0}^{\alpha } & Basal\;slip\;or\;extension\;twinning\\ \tau_{0}^{\alpha }exp(-T/\mu ) & Non-basal\;slip \end{array}\right.\;,$$

where μ is an empirical parameter to control the temperature effect and $\tau_{0}^{\alpha }$ stands for the reference slip resistance.

**Figure 2:**
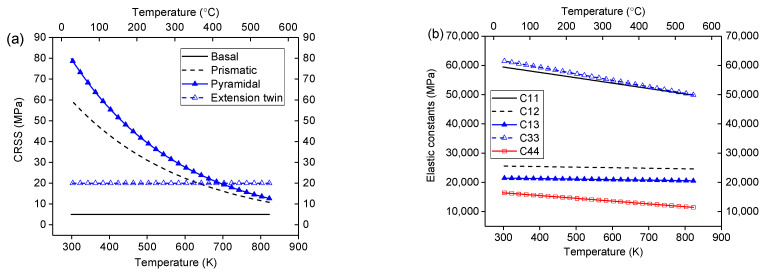
The evolution of parameters with respect to temperature, including CRSS (**a**) and elastic constants (**b**).

In the current work, the latent hardening effect is not considered due to the small strain amplitude encountered during heat treatments. Therefore, its influence on material behavior is assumed to be negligible. The material constants utilized in the study are set referring to experiment-based analysis [25] and theoretical computation [47]. They are listed in Table 1. Additionally, their variation with temperature is depicted in Figure 2.

Four deformation mechanisms are considered in the current work, including the basal $\left\{0001\right\}<11\bar{2}0>$ slip, the prismatic $\left\{10\bar{1}0\right\}<11\bar{2}0>$ slip, the <c+a> pyramidal $\left\{11\bar{2}2\right\}<11\bar{2}3>$ slip, and the $\left\{10\bar{1}2\right\}<10\bar{1}1>$ extension twin. All the systems are set to have the same rate sensitivity, m=0.05, which yields a good computation convergence and high efficiency [48]. According to previous studies [49,50], the Affine linearization overperforms other schemes and is employed in the current work.

The thermal expansion coefficients along the <a> and <c> directions are set as $\alpha_{a}=24.3\times 10^{-6}K^{-1}$ and $\alpha_{c}=27.1\times 10^{-6}K^{-1}$, respectively, which are referred to in literatures [51,52]. All the cooling processes are simulated starting from the condition of zero internal stress at a high initial temperature.

## 4. Results

### 4.1. Group I

(1) Stress response

**Figure 3:**
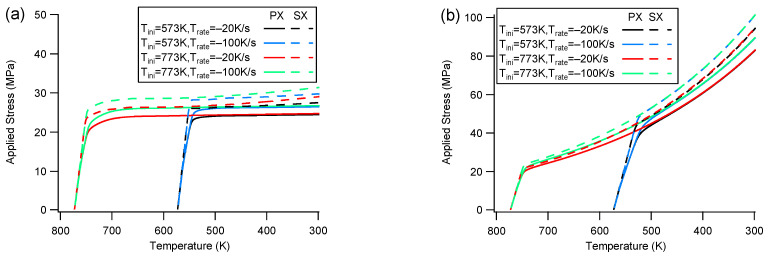
Comparison of applied stress evolution with temperature for rolled texture and single crystal under ND/<c>-constraint (**a**) and RD/<a>-constraint (**b**).

Figure 3 illustrates the stress response during cooling with constraint. The rolled texture and the single crystal exhibit similar behavior when the constraint is applied along the ND or <c> directions (Figure 3a). The only difference between the rolled texture and the single crystal is the magnitude of stress. The single crystal experiences higher applied stress compared to the polycrystal under the same cooling conditions. A distinct characteristic is observed, indicating that applied stress is established at the early cooling stage and remains relatively stable until the end. The initial temperature does not significantly affect the amplitude of applied stress, whereas a higher cooling rate leads to higher applied stress. This suggests that the dominating deformation mechanism may be temperature independent. Since the thermal strain rate is determined by the cooling rate when a thermal dilation tensor is given, a higher cooling rate results in a greater strain mismatch among the polycrystals. Consequently, the applied stress increases to accommodate the constrained boundary when the cooling rate is faster.

When the constraint is along the RD or <a> directions (Figure 3b), both the rolled texture and the single crystal exhibit a similar performance, with only small deviation in stress amplitude. The single crystal has a higher applied stress than the polycrystals. The applied stresses under RD/<a>-constraint continuously increase until the end. The yield stress for $T_{ini}=573\;K$ is higher than that for $T_{ini}=773\;K$, suggesting a potential temperature-dependent dominant deformation mechanism.

(2) Slip and twinning activities

**Figure 4:**
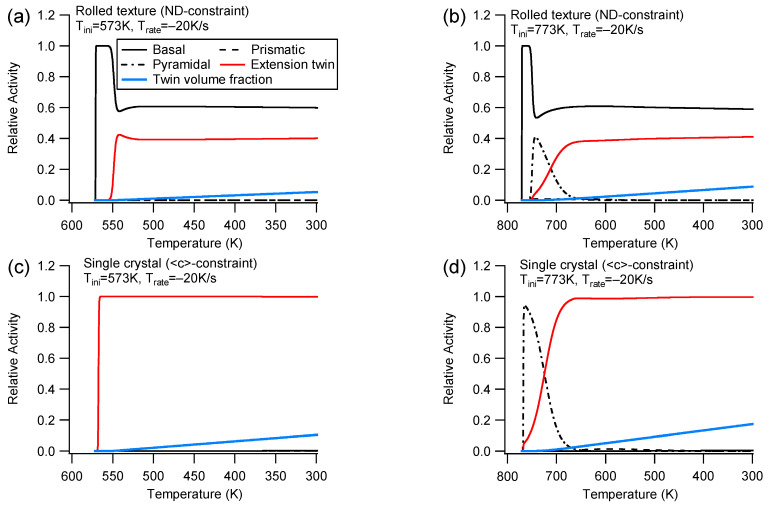
Relative activities of slips and extension twinning during cooling under the ND-constraint and $T_{rate}=-20\;K/s$ for the rolled texture (**a**,**b**) and the <c>-constraint for the single crystal (**c**,**d**) under different initial temperatures.

Figure 4 illustrates the slip and extension twinning activities during cooling at $T_{rate}=-20\;K/s$ for the ND/<c>-constraint. A close comparison of the slip/twinning activities with the counterpart for $T_{rate}=-100\;K/s$ shows that, under the same initial temperature, different cooling rates produce the same activity curve fashion and nearly identical relative activity values. This feature demonstrates that the slip and extension twinning relative activity is only influenced by the initial temperature during cooling with constraint. It should be noted that the relative activity only indicates the ratio of plastic strain for a deformation mode to the total plastic strain rather than the actual plastic strain amplitude for the deformation mode. Consequently, different deformation cases with the same slip relative activity may have different stress responses.

When cooling with the ND-constraint starting from $T_{ini}=573\;K$ (Figure 4a), the dominant modes of deformation are basal and extension twinning, which is similar to those observed under uniaxial tension along the ND. The basal slip is the only active system during the micro-yielding stage [12,53] at the beginning of cooling; then, the deformation transits to the active stage after yielding. This is because extension twinning has a high Schmid factor, while the basal slip has the lowest CRSS. In a single crystal with an ideal orientation, the basal slip exhibits a Schmid factor of zero. This indicates that only extension twinning is activated at $T_{ini}=573\;K$ (as shown in Figure 4c). Extension twinning governs the micro-yielding and the later stage. Consequently, the applied stress for $T_{ini}=573\;K$ in Figure 3a does not increase since both basal slip and extension twinning are temperature independent.

Unlike $T_{ini}=573\;K$, where there is no active pyramidal activity during the micro-yielding stage in the early cooling process (Figure 4a), $T_{ini}=773\;K$ exhibits active pyramidal activity (Figure 4b) as the pyramidal CRSS with a high Schmid factor at high temperature becomes low enough to be activated. Since single crystals have an ideal orientation where the c-axis is parallel to the constraint direction, the basal slip is not allowed to occur. However, due to the low CRSS of the pyramidal slip, which is even lower than that of extension twinning, only the pyramidal slip and extension twinning can be activated during micro-yielding and post-yielding stages under $T_{ini}=773\;K$ for the single crystal (Figure 4d). Although there is a short duration of the pyramidal slip with low CRSS at high temperature, no remarkable increase in applied stress can be found under $T_{ini}=773\;K$ (refer to Figure 3a) since the soft pyramidal slip accommodates part of the thermal strain mismatch.

**Figure 5:**
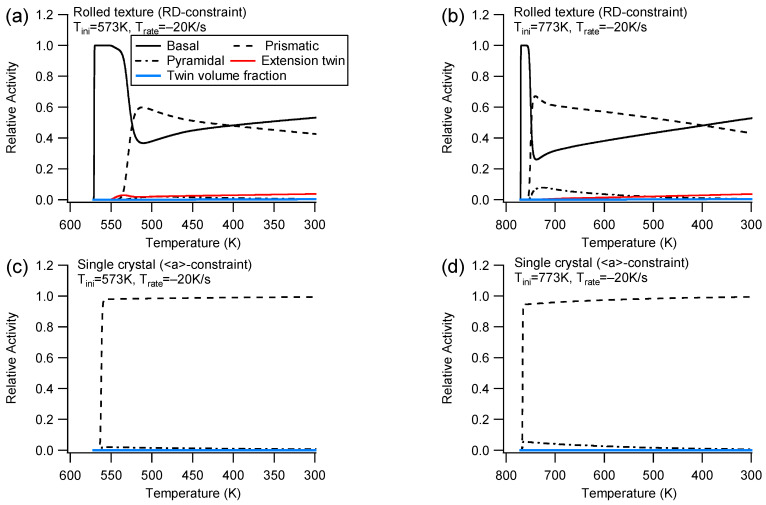
Relative activities of slips and extension twinning during cooling with RD-constraint and $T_{rate}=-20\;K/s$ for rolled texture (**a**,**b**) and <a>-constraint for a single crystal (**c**,**d**) at different initial temperatures.

Figure 5 illustrates the activities of deformation mechanisms under a cooling rate of $T_{rate}=-20\;K/s$ for the rolled texture and the single crystal with the RD/<a>-constraint. A comparison of the relative activities with the counterpart for $T_{rate}=-100\;K/s$ reveals that, regardless of the cooling rate, slip relative activities remain unaffected when the initial temperature is held constant. Similar to cooling with the ND/<c>-constraint, the slip relative activities can only be changed by the initial temperature.

Under $T_{ini}=573\;K$, the activities of deformation mechanisms for the rolled texture are similar to those of tension along the RD (Figure 5a). The primary deformation mechanisms are the basal and prismatic slips. The basal slip governs the micro-yielding stage, while the prismatic slip additionally contributes to the domination during post-yielding. A single crystal with the <a>-constraint only exhibits an active prismatic slip (Figure 5c) because at least two prismatic slip variants have a non-zero Schmid factor, while the basal plane is parallel to the constraint direction.

Under $T_{ini}=773\;K$, an additional noticeable pyramidal slip can be observed in both the rolled texture and the single crystal (Figure 5b,d). The activation of the pyramidal slip is attributed to its low CRSS at high temperatures. The occurrence of the pyramidal slip further reduces the activity of the basal slip or the prismatic slip in the rolled texture (Figure 5a) or the single crystal (Figure 5c), respectively.

(3) Elastic lattice strain

**Figure 6:**
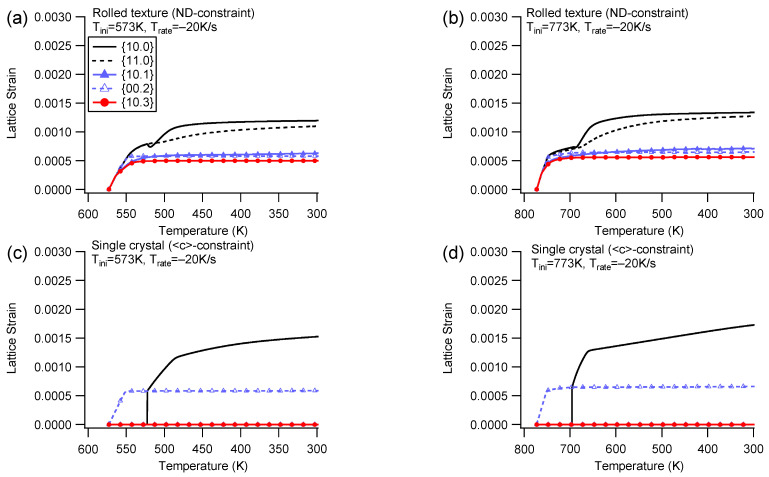
Lattice strain of different diffraction planes during cooling with the ND-constraint and $T_{rate}=-20\;K/s$ for the rolled texture (**a**,**b**) and the <c>-constraint for the single crystal (**c**,**d**) under different initial temperatures. The lattice strain is detected along the ND/<c>.

Figure 6 illustrates the evolution of elastic lattice strain during cooling with the ND/<c>-constraint under $T_{rate}=-20\;K/s$. Additionally, a detailed examination of the lattice strain evolution at $T_{rate}=-100\;K/s$ is also carried out, showing similar patterns to that of $T_{rate}=-20\;K/s$. The elastic lattice strain is detected along the constraint direction to simulate neutron diffraction measurements. A deviation angle of 7.5° is utilized to determine whether a diffraction plane belongs to a grain family. The lattice strain depicted in the figure represents an average value among the grain family. This processing method for the lattice strain is subsequently applied in this study.

In the rolled texture, the ND-constraint generates tensile strain for the observed grain families (Figure 6a,b). During the early elastic stage, all the grain families exhibit a nearly linear increase in lattice strain. This is followed by micro-yielding, which begins at the bifurcation of elastic lattice strains, and a post-yielding stage that starts from notable yielding and approaching saturation. The linear stage implies elastic isotropy during the very early stages of cooling. The {10.0} and {11.0} grain families exhibit the highest tensile strain, indicating that they are the hardest orientations. These two grain families have prismatic planes that are either perpendicular or parallel to the ND, making both basal slip and extension twinning impossible. Due to a high CRSS at low temperatures, two prismatic variants can possibly be activated, which allows the {10.0} and {11.0} grain families retention of a high elastic lattice strain, regardless of the extent of prismatic slip activity. The {00.2} grain family favors extension twinning, resulting in low lattice strain. The {10.3} and {10.1} grain families have basal planes inclining 58° and 28.1° to the ND, respectively, making them prone to trigger the basal slip. Therefore, the {00.2}, {10.3}, and {10.1} planes experience low lattice strain.

The single crystal only possesses two grain families with positive lattice strain (Figure 6c,d). There are no child grain families such as {11.0}, {10.1}, and {10.3} among the reorientated regions, except for {10.0}. The {00.2} grain family favors extension twinning and retains low lattice strain, while the {10.0} grain family is formed with a low volume fraction (maximum ~10%, as shown in Figure 4c) and retains high lattice strain. Due to fewer orientations present in the sample, the single crystal has limited ability to accommodate the constraint boundary, resulting in a higher applied stress amplitude compared to the rolled texture counterpart (Figure 3a). Another contributing factor to the higher applied stress in the single crystal could be its greater contraction compared to that of the polycrystals, as orientations other than the {00.2} grain family exhibit less contraction. As a result, the polycrystals experience less contraction than the single crystal, leading to a lower applied stress level.

In correspondence with the evolving fashion of the applied stress in Figure 3a, the lattice strain is also established within the early range of ~100 K. This phenomenon can be attributed to the energy induced by thermal anisotropy dissipating during plastic deformation. As the energy is dissipated, the energy required for driving plastic deformation decreases until the elastic lattice strains stabilize and approach equibrium.

**Figure 7:**
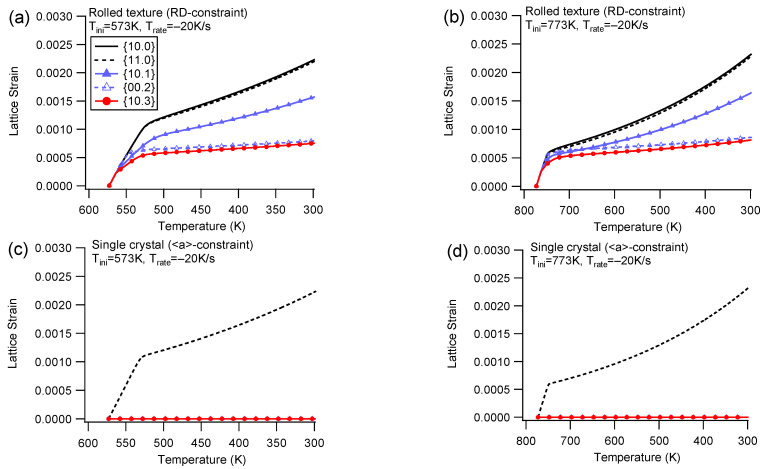
Lattice strain of different diffraction planes during cooling with the RD-constraint and $T_{rate}=-20\;K/s$ for the rolled texture (**a**,**b**) and the <a>-constraint for the single crystal (**c**,**d**) under different initial temperatures.

Figure 7 illustrates the evolution of elastic lattice strain during cooling with the RD/<a>-constraint at $T_{rate}=-20\;K/s$. An additional close examination of the lattice strain evolution for the counterpart at $T_{rate}=-100\;K/s$ reveals that the pattern of lattice strain evolution is similar to that at $T_{rate}=-20\;K/s$.

For the rolled texture (Figure 7a,b), the {10.0} and {11.0} grain families exhibit a preference for the prismatic slip, resulting in the highest lattice strain being shared by them. The {10.3} grain family, on the other hand, predominantly favors the basal slip, leading to a low lattice strain. In comparison to the low lattice strain amplitude observed for the rolled texture with the ND-constraint, the {10.1} grain family displays a relative high lattice strain. This difference can be attributed to the internal interaction of strain accommodation, where the {10.1} grain family experiences distinct stress conditions between the ND-constraint and the RD-constraint.

Different from the rolled texture, the single crystal only possesses one grain family of {11.0} with a positive lattice strain (Figure 7c,d). The single crystal solely relies on the prismatic slip to accommodate the constrained boundary, while the rolled texture predominantly consists of a grain family of {10.0}, which favors the prismatic slip, as well as other orientations that favor the basal slip. Consequently, the single crystal exhibits a higher applied stress amplitude compared to its rolled texture counterpart.

Table 2 summarizes the final lattice strains for each diffraction plane under different cooling conditions in Group I. Internal stress is calculated by multiplying the lattice strain with Young’s modulus (approximately 45 GPa). A notable observation is that when the initial temperature is the same, a higher cooling rate results in higher applied stress, regardless of whether it is for the rolled texture or the single crystal. A higher cooling rate generates a higher thermal strain rate, which induces a higher internal stress under the rate-dependent framework. Therefore, under cooling with constraint, a slightly higher applied stress responds to the higher cooling rate.

The lattice strain under cooling with constraint indicates that all diffraction planes detected along the constraint direction undergo tensile lattice strain that corresponds to the tensile stress in the constraint boundary. Conversely, if compressive stress is applied, all detected diffraction planes can undergo compressive lattice strain [19]. Thus, when an external load is applied, all diffraction planes along the loading direction must coordinate with it.

### 4.2. Group II

(1) Slip and twinning activities

**Figure 8:**
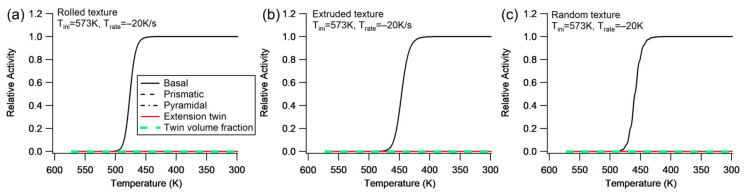
Evolution of slip and extension twinning activity during cooling without constraints at $T_{rate}=-20\;K/s$ for the rolled texture (**a**), extruded texture (**b**), and random texture (**c**). The activity curves of prismatic slip, pyramidal slip, and extension twinning are overlapping.

Figure 8 illustrates the activity of each deformation mechanism for different textures under cooling without constraint at $T_{rate}=-20\;K/s$. The only active slip system is basal slip, suggesting that the elastic energy resulting from thermal anisotropy may not be sufficient to initiate higher CRSS deformation mechanisms. Additional analysis reveals that the slip activities are the same under a higher cooling rate ($T_{rate}=-100\;K/s$) as shown in Figure 8. Similar to the cooling process with constraint, the cooling rate without constraint does not affect the slip and twinning activities for these three types of textures. Additionally, a higher initial temperature also results in the basal slip being the sole active system, following a similar pattern as shown in Figure 8.

(2) Elastic lattice strain

**Figure 9:**
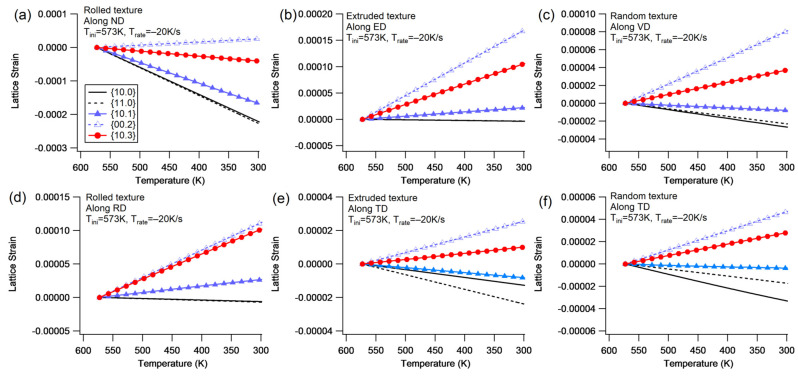
Lattice strain evolution during cooling without constraint for the rolled texture (**a**,**d**), extruded texture (**b**,**e**), and random texture (**c**,**f**) (note that the vertical axis ranges are different from each other).

Figure 9 shows the evolution of elastic lattice strain under cooling from $T_{ini}=573\;K$ at $T_{rate}=-20\;K/s$ without constraint for the three types of textures. The lattice strains for the three types of textures exhibit different characteristics along the two orthogonal directions. Generally, the rolled texture tends to experience tensile strain along the RD and compressive strain along the ND. On the other hand, the extruded texture tends to undergo tensile strain along the ED and compressive strain along the TD. The random texture tends to exhibit uniform tensile and compressive strain on diffraction planes along the VD or TD. Figure 9 implies that the basal slip is very limited, as the lattice strain of each diffraction plane evolves linearly without any inflection point caused by significant plastic deformation.

**Figure 10:**
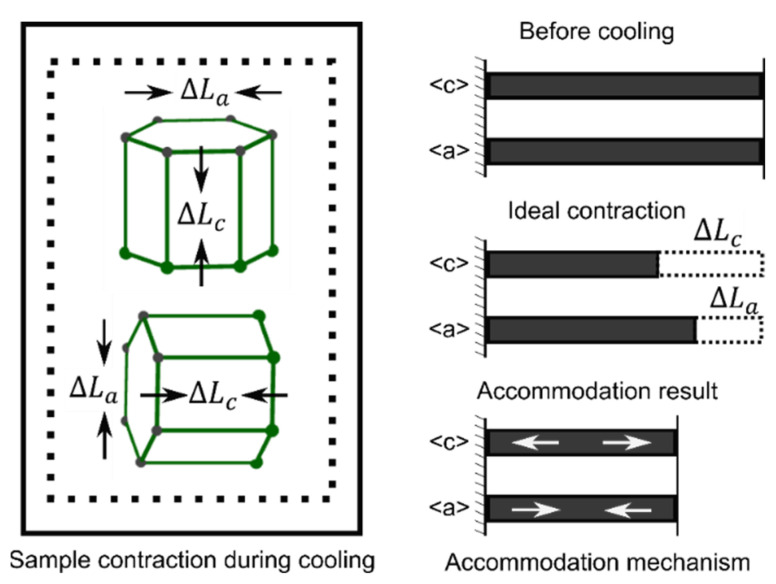
Schematic diagram of strain accommodation mechanism during cooling.

For the rolled texture, the dimension along the ND experiences greater contraction compared to that along the RD. This is due to the fact that the {00.2} grain family constitutes the majority of the polycrystals and undergoes larger thermal contraction along the <c> direction than along the <a> direction. Consequently, along the ND, the {00.2} grain family undergoes tensile strain, while other grain families experience compressive strain (refer to Figure 9a). The accommodation mechanism is schematically illustrated in Figure 10. Regarding the RD, both the {11.0} and {10.0} grain families exhibit compressive strain, as indicated by the simulation result shown in Figure 9d. When observed along the RD, the majority grain families, namely {11.0} and {10.0}, contract to a lesser extent than the {00.2} grain family. In order to balance with the thermal strain mismatch, the minor grain families with a smaller volume fraction need to contribute more elastic strain. Conversely, the majority of the grain families with a larger volume fraction only need to offer a small amount of elastic strain to balance with other grains. Consequently, the other minor grain families experience tensile strain, albeit with a higher amplitude than that experienced by the {11.0} and {10.0} grain families.

For the extruded texture, the majority of the polycrystals, when detected along the ED, are the {11.0} and {10.0} grain families. These two grain families exhibit less contraction along the ED compared to other orientations, resulting in compressive strain (shown in Figure 9b). The minor grain families with a smaller volume fraction experience larger tensile strain. However, the lattice strains detected along the TD are more uniformly distributed for the diffraction planes (Figure 9e). The maximum tensile strain exhibits a similar amplitude to that of the maximum compressive strain. This feature can be attributed to the random distribution of c-axes around the ED, causing the majority {11.0} and {10.0} grain families detected along the ED to scatter uniformly as each grain family detected along the TD. Furthermore, the uniform distribution of orientations detected along the TD results in a lower residual strain amplitude compared to that detected along the ED and the rolled texture as well. This feature also demonstrates that randomly distributed orientations can mitigate the strain mismatch caused by the thermal anisotropy of the single crystal.

For the random texture (Figure 9c,f), the detected residual strain amplitude along both the VD and TD on each diffraction plane is significantly lower and more uniform than that of the rolled texture (Figure 9a) and the extruded texture (Figure 9b). The presence of the random texture results in mechanical isotropy in the mesoscale for the polycrystals, which is due to the thermal isotropy at the scale. The thermal anisotropy between the <c> and <a> directions is effectively averaged to a negligible level in the polycrystals with random orientations. As a result, in the mesoscale, there is no noticeable difference in thermal contraction between different directions. The slightly higher lattice strain of {00.2} (Figure 9c) is attributed to the non-ideal and spatially non-uniform orientation distribution in the random texture. This slight nonuniformity causes the {00.2} grain family to exhibit a slightly higher or lower lattice strain amplitude than the average level shown in Figure 9f.

Table 3 presents the residual lattice strain for the three typical textures after cooling without constraint under different cooling conditions. Several general features can be observed: (i) The maximum amplitude of residual lattice strain differs among the textures in the following order from high to low: rolled > extruded > random. This suggests that the rolled texture exhibits stronger thermal anisotropy than the extruded texture at the mesoscale, while the random texture demonstrates the least thermal anisotropy. (ii) Contrary to the findings in Section 4.1 for cooling with constraint, higher cooling rates do not result in higher residual lattice strain during cooling without constraint. Although a higher cooling rate leads to a greater thermal strain rate under cooling without constraint, all grains contract simultaneously only through interaction with each other. It is possible for the polycrystals to counteract the influence of the thermal strain rate without the intervention of boundary constraints. (iii) A higher initial temperature under cooling without constraint yields a higher residual lattice strain. This may be attributed to the longer cooling process from a higher initial temperature to room temperature, which generates a greater amount of thermal strain energy due to thermal anisotropy. Since there is no significant plastic deformation, most strain mismatch is transformed to reserved lattice strain among the polycrystals. (iv) All the {00.2} grain families detected in any direction undergo tensile strain, while the {11.0} and {10.0} grain families experience compressive strain, as expected according to the forementioned analogical analysis.

## 5. Discussion

### 5.1. Influence of Thermal Anisotropy for the Single Crystal

**Figure 11:**
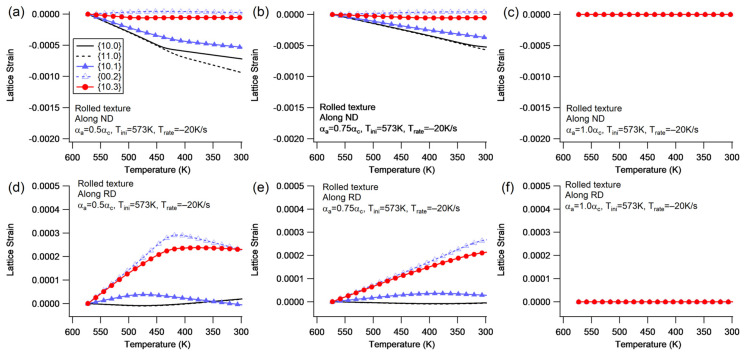
The evolution of lattice strain for the rolled texture during cooling without constraint at different $\alpha_{a}/\alpha_{c}$ ratios: 0.5 (**a**,**d**), 0.75 (**b**,**e**), and 1.0 (**c**,**f**).

The thermal anisotropy of the single crystal is the primary source of the internal strain mismatch and residual internal stress. The analysis of the cooling processes with and without constraint suggests that the initial temperature and cooling rate could impact the generation of internal stress. In order to determine the influence of the thermal anisotropy of a single crystal on the mesoscale mechanical anisotropy, an additional simulation was conducted. Figure 11 illustrates the evolution of lattice strain for various $\alpha_{a}/\alpha_{c}$ values.

When $\alpha_{a}/\alpha_{c}=0.5$, the elastic lattice strain increases to a high enough level to trigger more plastic deformation. In addition to the basal slip, limited extension twinning is observed after 435 K. This can be seen by the presence of an inflection point on the {00.2} plane detected along the RD in Figure 11d. However, no noticeable inflection point is observed on the {00.2} grain family detected along the ND, as the majority {00.2} grain family is difficult to trigger extension twinning. On the other hand, the {10.0} grain family detected along the ND favors prismatic slip, but its lattice strain deviates from that of the {11.0} grain family due to the compressive strain undertaken by the newly reoriented {10.0} grain family. When examining along the RD, the lattice strain across the {00.2} plane undergoes an inflection point with an amplitude of 0.000282 (12.7 MPa) (Figure 11d). This is significantly lower than the applied stress under uniaxial tension (40 MPa). The lower applied stress across the basal plane may also be influenced by the complex stress state. In such a state, interactions between grains involve various stress components beyond normal stress, which may promote the activation of extension twinning. Moreover, the {10.1} diffraction plane detected along the RD exhibits an inflection point at ~476 ℃. This is attributed to the occurrence of the basal slip with lower CRSS, resulting in a lower lattice strain.

When $\alpha_{a}/\alpha_{c}$ increases, the thermal anisotropy of the single crystal decreases, and the plastic deformation is reduced, resulting in a delayed bifurcation for {10.0}, as well as a lower residual lattice strain (Figure 11b,e). When $\alpha_{a}/\alpha_{c}=1.0$, the thermal anisotropy disappears, and no internal strain mismatch occurs (Figure 11c,f). The Mg alloy exhibits less significant thermal anisotropy compared to Zircaloy-2 due to its higher ratio (~0.90) of $\alpha_{a}/\alpha_{c}$, which is higher than that of Zircaloy-2 (~0.5) [9,54,55]. The limited thermal anisotropy of the Mg alloy prevents the generation of high internal strain during cooling without constraint, resulting in only restricted basal slip and linear lattice strain evolution.

In light of this, the thermal residual stress induced by intergranular mismatch in the thermal strains plays a significant role that should not be overlooked in subsequent loading after heat treatments for HCP materials. The tensile strain along the <c> direction and the compressive strain along the <a> direction for the Mg alloy are generated during cooling, which is consistent with the findings of neutron diffraction measurements for Zirconium alloys [9,56]. The tensile residual stress along the <c> direction promotes extensive extension twinning at the early stage of subsequent tension along the ND [55]. As a result, it is recommended to further investigate the microscale influence of thermal residual stress on the post-cooling strength [34] and fracture [57] with careful attention.

### 5.2. The Role of Intrinsic Thermal Stress in Macroscale Cooling

The mesoscale simulation of cooling without constraint by the self-consistent model captures the thermal stress induced only by thermal anisotropy of the single crystal and the grain–grain interaction, without the interference of the macroscale dimension. Consequently, the thermal stress produced in this ideal theoretical approach reflects the fundamental property of thermal–mechanical behavior, which could be referred to as intrinsic thermal stress.

When cooling without constraint, the thermal elastic strain energy, cooling time and initial temperature are all positively correlated, independent of the cooling rate. However, during cooling with constraint, either a higher initial temperature or a faster cooling rate can result in greater applied stress along the constraint direction. In the practical structural components undergoing macroscale cooling, there is a temperature gradient between the surface and the interior of the part, which leads to compressive stress on the surface and tensile stress in the internal region. Due to this, the constraint condition during cooling cannot be regarded as an ideal state where the RD or the ND are constrained as presented in Section 4.1. As a hypothesis, we speculate that the macroscopic thermal stress is the sum of the intrinsic thermal stress and additional stress induced by a macroscale strain mismatch.

The intrinsic thermal stress can only be influenced by the thermal property of the single crystal (i.e., thermal expansion coefficients along the <c> and <a> directions), as well as its texture features. Conversely, the shape and dimensions of the sample structure solely affect the additional stress. Based on the low stress amplitude in Section 4.2 compared to the practical quench residual stress, which is several tens of megapascals [34], it is evident that the additional stress exhibits a greater magnitude than the intrinsic thermal stress. The distribution of residual stress is chiefly influenced by the macroscale strain mismatch. The hypothesis of the superposition of intrinsic and additional stresses can further provide insights into subtle mechanical phenomena that were not well understood in previous observations.

Taking a practical case as an example, it can be observed that the surface of a square thin plate with an extruded texture experiences higher compressive stress ($\sigma_{xx}$) along the ED (in the plate plane) compared to that ($\sigma_{yy}$) along the long transverse direction (LTD) after quenching [34]. Based on the strain accommodation mechanism during cooling as explained in Section 4.2, in the absence of a temperature gradient, the ED coinciding with the <a> direction of the majority grains experiences intrinsic compressive stress ($\sigma_{xx}^{intr}$), while the LTD experiences slight stress ($\sigma_{yy}^{intr}$). If the thermal expansion anisotropy of a single crystal is not considered, the symmetric shape of the square plate results in additional compressive residual stress along the ED ($\sigma_{xx}^{add}$), which is the same as that along the LTD ($\sigma_{yy}^{add}$). When the thermal expansion anisotropy of a single crystal is taken into account, the superposition of $\sigma_{xx}^{intr}$ and $\sigma_{xx}^{add}$ is greater than that of $\sigma_{yy}^{intr}$ and $\sigma_{yy}^{add}$. Moreover, the compression along the ED greatly promotes extension twinning, thereby further reducing $\sigma_{xx}$.

The strain accommodation mechanism during cooling can also be applied to the friction–stir welding process for magnesium alloys. For a rolled aluminum alloy with a more symmetric crystal structure, the tensile residual stress along the WD ($\sigma_{xx}$) behaves as an “M” shape from the advancing side to the retrieving side across the centerline of the SZ in the middle plane [58]. However, for a rolled magnesium alloy, $\sigma_{xx}$ encounters severe fluctuation near the transition zone (TZ) and in the SZ [59]. Previous observations have reported a strong texture in the SZ, where the basal planes trace an ellipsoidal surface surrounding the centerline of the rotating pin [59,60,61,62,63]. The rotating pin causes severe plastic flow, resulting in a gradual transition of the *c*-axes orientation from the WD in the SZ to the TD in the TZ. Strong texture discrepancy can be found from the SZ to the TZ, and it extends to the base material (BM), resulting in notable localized strain accommodation and stress fluctuations.

Another feature of $\sigma_{xx}$ in a post-welded rolled texture plate is that the value in the grains with *c*-axes parallel to the WD ($\sigma_{xx}^{c}$) is higher than that in the grains with *a*-axes parallel to the WD ($\sigma_{xx}^{a}$) in the SZ [64]. The *c*-axes in the center of SZ are oriented roughly near the WD, demonstrating a strong B-fiber texture analogous to the rolled texture. Based on the strain accommodation mechanism during cooling, the intrinsic part of $\sigma_{xx}^{c}$ is positive, while the corresponding part for $\sigma_{xx}^{a}$ is negative. When additional tensile residual stress is superposed, $\sigma_{xx}^{c}$ becomes higher than $\sigma_{xx}^{a}$.

## 6. Conclusions

The thermal stress induced by the thermal anisotropy of a single crystal is examined via a thermal elastic viscoplastic self-consistent model (EVPSC). This model accounts for temperature-dependent elastic constants and the critical resolved shear stress and thermal dilation tensor. Cooling processes with and without constraint are simulated for rolled texture samples, a single crystal, extruded texture samples, and randomly oriented texture samples. From these simulations, the following conclusions can be drawn:

(1) The elastic lattice strain is significantly higher under cooling with constraint compared to that under cooling without constraint. The deformation mechanism observed under cooling with constraint resembles tension along the direction of constraint at room temperature. Polycrystals offer more deformation mechanisms to accommodate the thermal anisotropy, leading to a lower applied stress within the constrained boundary, in contrast to the single crystal.
(2) Among the observed textures, the maximum amplitude of residual lattice strain follows this order, from high to low: rolled > extruded > random. The overall polycrystals exhibit lower internal stress due to reduced thermal anisotropy. In the rolled texture, the {10.0} and {11.0} planes experience the highest magnitude of compressive lattice strain, while the extruded texture demonstrates the highest magnitude of tensile lattice strain on the {00.2} plane.
(3) The strain accommodation mechanism during cooling without constraint can be captured by the thermal EVPSC model, in which the intrinsic thermal strain is solely derived from the interactions between grains. As the cooling process progresses, a single crystal within aggregates contracts more prominently along the <c> direction compared to the <a> direction. Consequently, the {00.2} plane undergoes tensile lattice strain, while the {10.0} and {11.0} planes experience compressive lattice strain.
(4) A speculation is proposed that the macroscopic thermal stress is the sum of the intrinsic thermal stress and the additional stress induced by a macroscale strain mismatch. Based on this hypothesis and the strain accommodation mechanism during cooling, a deeper understanding of the anisotropic residual stress can be promoted to macroscale cooling.

## Figures and Tables

**Table 1:** Model constants used in the study.

Mode	$\tau_{0}$ (MPa)	μ	Elastic Constant	$C_{ij}^{0}$ (MPa)	$p_{ij}$ (MPa)	$q_{ij}$
Basal	5	-	$C_{11}$	6.34 × 10^4^	3.577 × 10^3^	192.6
Prismatic	158.7	306.5	$C_{12}$	2.59 × 10^4^	0.661 × 10^3^	339.6
Pyramidal	227.9	284.8	$C_{13}$	2.17 × 10^4^	0.857 × 10^3^	458.4
Extension twin	20	-	$C_{33}$	6.64 × 10^4^	4.282 × 10^3^	190.2
			$C_{44}$	1.84 × 10^4^	2.125 × 10^3^	219.9

**Table 2:** Residual internal stress on different crystalline planes under different cooling conditions for the rolled texture and single crystal (unit: MPa, the maximum value is marked in bold).

Constraint Direction	Initial Temp.	Temp. Rate	{10.0}	{11.0}	{10.1}	{00.2}	{10.3}
ND	300	−20	**53.9**	49.6	28.4	26.2	22.6
<c>			**68.7**	0	0	26.5	0
ND	300	−100	**58.1**	53.3	28.3	28.3	24.3
<c>			**73.4**	0	0	28.5	0
ND	500	−20	**60.2**	57.5	32.0	29.3	25.4
<c>			**77.9**	0	0	29.7	0
ND	500	−100	**64.8**	61.9	34.4	31.5	27.0
<c>			**83.6**	0	0	32.0	0
RD	300	−20	**105.8**	99.2	70.8	35.8	33.9
<a>			0	**106.7**	0	0	0
RD	300	−100	**108.5**	107.0	76.4	38.6	36.5
<a>			0	**108.5**	0	0	0
RD	500	−20	**104.6**	103.1	74.0	38.8	36.7
<a>			0	**104.6**	0	0	0
RD	500	−100	**112.8**	111.2	79.7	41.8	39.4
<a>			0	**112.6**	0	0	0

**Table 3:** Residual internal stress on different crystalline planes for the rolled, extruded, and random textures after cooling without constraint under different conditions (unit: MPa, the maximum value is marked in bold).

Tex.	Ini. Temp./ Temp. Rate	Dir.	{10.0}	{11.0}	{10.1}	{00.2}	{10.3}
Rolled	300/−20	ND	−10.0	**−10.3**	−7.6	1.2	−1.8
		RD	−0.3	−0.3	1.2	**5.0**	4.6
Rolled	300/−100	ND	−10.0	**−10.3**	−7.6	1.2	−1.8
		RD	−0.3	−0.3	1.2	**5.0**	4.6
Rolled	500/−20	ND	−17.6	**−18.0**	−12.7	1.8	−2.6
		RD	−0.4	−0.4	1.7	**8.9**	7.7
Rolled	500/−100	ND	−17.5	**−17.9**	−12.9	1.8	−2.8
		RD	−0.4	−0.5	1.8	**8.8**	7.7
Extruded	300/−20	ED	−0.2	−0.2	1.0	**7.7**	4.8
		TD	−0.6	−1.1	−0.4	**1.2**	0.5
Extruded	300/−100	ED	−0.2	−0.2	1.0	**7.6**	4.7
		TD	−1.4	**−1.8**	−0.9	1.4	0.7
Extruded	500/−20	ED	−0.2	−0.2	1.7	**13.2**	7.9
		TD	−2.4	**−3.1**	−1.6	2.4	1.1
Extruded	500/−100	ED	−0.2	−0.2	1.7	**13.3**	8.1
		TD	−2.5	**−3.1**	−1.6	2.4	1.1
Random	300/−20	VD	−1.2	−1.0	−1.0	**3.6**	1.7
		TD	−1.5	−0.8	−0.2	**2.1**	1.3
Random	300/−100	VD	−1.2	−1.0	−0.3	**3.6**	1.7
		TD	**−1.5**	−1.4	0.0	1.2	1.1
Random	500/−20	VD	−2.0	−1.7	−0.6	**6.3**	2.9
		TD	**−2.5**	−2.5	0.1	2.1	2.0
Random	500/−100	VD	−2.1	−1.8	−0.6	**6.3**	2.9
		TD	**−2.5**	−2.4	0.0	2.1	2.0

## Data Availability

The data presented in this study are available on request from the corresponding author.
